# HIV self-testing: A highly acceptable and feasible strategy for reconnecting street adolescents with HIV screening and prevention services in Togo (The STADOS study)

**DOI:** 10.1371/journal.pone.0312693

**Published:** 2024-10-24

**Authors:** Arnold Junior Sadio, Harold Régis Kouanfack, Rodion Yao Konu, Fifonsi Adjidossi Gbeasor-Komlanvi, Gagnon Kwami Azialey, Herbert Kokou Gounon, Martin Kouame Tchankoni, Amivi Phyllis Amenyah-Ehlan, Anoumou Claver Dagnra, Didier Koumavi Ekouevi

**Affiliations:** 1 Faculty of Health Sciences, Department of Public Health, University of Lomé, Center for Training and Research in Public Health, Lomé, Togo; 2 African Center for Research in Epidemiology and Public Health (CARESP), Lomé, Togo; 3 National Institute for Health and Medical Research (INSERM), Research Institute for Sustainable Development (IRD), University of Bordeaux, Bordeaux Population Health Centre, UMR 1219, Bordeaux, France; 4 HIV Management and Co-infections, Health District Administration, Yoto, Togo; 5 University of Lomé, Laboratory of Molecular Biology and Immunology, Lomé, Togo; 6 Ministry of Health, National Program for the Fight against HIV, Viral Hepatitis and Sexually Transmitted Infections, Lomé, Togo; University of Bunia; Centre Interdisciplinaire de Recherche Translationnelle en Medecine et Sciences de la Sante (CIRTMSS), THE DEMOCRATIC REPUBLIC OF THE CONGO

## Abstract

**Introduction:**

HIV self-testing is a complementary screening strategy that could facilitate access to HIV care services for street adolescents. The objectives of this study were to assess the acceptability and feasibility of HIV self-testing and their associated factors, to estimate HIV prevalence among street adolescents in Togo, and to describe the sexual behavior of this population.

**Methods:**

A cross-sectional study was conducted between July 2021 and May 2022 in Lomé and Kara (Togolese cities with the highest number of street adolescents). Street adolescents aged 13–19 years were included. An oral HIV self-test (OraQuick®) was used. Acceptability was defined as the proportion of adolescents who completed the test, and feasibility was defined as the proportion of adolescents who reported a test with a valid result. An HIV serological test was performed for all participants. A weighted logistic regression model was used to identify the factors associated with the acceptability and feasibility of HIV self-testing.

**Results:**

A total of 432 street adolescents (12.3% female) with a median age of 15 years, interquartile range (IQR) [14–17], were included in this study. Of the 231 sexually active adolescents, only 30.3% (n = 70) reported having used a condom during their last sexual intercourse. HIV self-test was offered to a sub-sample of 294 street adolescents. Acceptability was 96.6% (284/294), (95%CI = [93.8–98.3]) and feasibility 98.9% (281/284), (95%CI = [97.0–100.0]). Being 16 years of age or older (aOR = 28.84; p<0.001) was associated with HIV self-test acceptability. Reporting drug abuse (aOR = 0.47; p = 0.020) was negatively associated to acceptability. Having an educational level at least equivalent to secondary school was associated to HIV self-testing feasibility (aOR = 3.92; p = 0.040). Self-test results were correctly interpreted by 98.6% of street adolescents. HIV prevalence was estimated at 0.9% (95%CI [0.4–2.4]).

**Conclusion:**

HIV self-testing is acceptable and feasible among street adolescents, a population at high risk of HIV infection in Togo. The provision of HIV self-testing kits, coupled with condom distribution, represents an opportunity to improve access to HIV care services.

## Introduction

Adolescents are a vulnerable population particularly exposed to HIV [1]. According to the United Nations Children’s Fund (UNICEF) adolescents and young people constitute a growing proportion of individuals living with HIV worldwide. In 2023 alone, 360,000 young people aged 15 to 24 were newly infected with HIV, including 140,000 adolescents aged 15 to 19 [2]. In addition, this population has been reported to have limited access to the healthcare system, in particular to HIV testing and support services [3,4]. HIV testing, as a means of accessing healthcare, remains particularly inadequate among adolescents living in sub-Saharan Africa. In fact, less than 20% of adolescents aged 15 to 19 living in West and Central Africa are aware of their HIV status [5].

Street children differ from other young people in that they are the most exposed to the risk of HIV infection, whether through their sexual behavior or drug use [6]. Street children are defined as children who depend on the street to live and/or work, who have a strong link with public spaces (e.g. streets, markets, parks, bus or train stations) and for whom the street plays an essential role in their daily life and identity [7]. Risky sexual behavior among street adolescents is generally marked by early sexual activity, inconsistent condom use, multiple sexual partners and survival sex (sex in exchange for money, food and protection) [8–10]. Furthermore, most of these adolescents have limited knowledge of HIV and sexually transmitted infections (STIs) [8,11,12]. This lack of knowledge and the resulting risky behaviors increase the risk of HIV and STIs spreading among this population.

According to the World Health Organization’s (WHO) unified guidelines on HIV testing, young people from vulnerable populations such as street children should be prioritized for HIV testing [13]. To improve access to HIV testing services for these groups, the WHO recommends HIV self-testing [13]. Existing data show that HIV self-testing is acceptable and feasible in a wide variety of populations, and that social harm (stigma, discrimination) associated with self-testing is rare [14]. HIV self-testing is an innovative tool that promotes patient empowerment and contributes to the achievement of the first “95” of the United Nations 95-95-95 targets [13,15]. However, while many countries in Sub-Saharan Africa are integrating self-testing into their screening strategies, there are few studies on the acceptability and feasibility of HIV self-testing among adolescents [16–18]. Also, no published study in sub-Saharan Africa has evaluated this strategy among street adolescents and data on HIV prevalence in this specific population remains limited in sub-Saharan Africa. In Togo, in late 2021, with support from PEPFAR and FHI 360, the country launched a pilot phase of self-testing implementation in 13 care sites. The target populations were people in the general population over the age of 15, sex workers, and men who have sex with men. However, street youth were not included, and no acceptability and feasibility studies were conducted prior to this pilot phase [19]. The aim of this study was to assess the acceptability and feasibility of HIV self-testing and their associated factors, to estimate HIV prevalence among street adolescents in Togo, and to describe the sexual behavior of this population.

## Methods

### Study design and period

A cross-sectional study (The Street Adolescents Survey—StAdoS) was carried out between July 2021 and May 2022 in Lomé, the capital city in the south of Togo, and Kara, the second most affluent city in the north of Togo [20]. More than 60% of Togo’s street adolescents live in these two cities [21].

### Study population and sampling

The study involved four local non-governmental organizations (NGOs) that provide social support (basic care, security) to street adolescents: ANGE, AGOPODE, JADE and CREUSET-Togo.

All male and female street adolescents aged between 13 and 19 who had been living on the street for at least three months were eligible for the study. Each street adolescent who met the inclusion criteria was invited to participate in the study and was included if they gave their consent. However, given the specificities of the street adolescent population, we had to adapt our recruitment method. In fact, street adolescents in Togo are structured into very supportive groups, and each group has leaders. They protect each other and the notion of trust is a collective one. In other words, either "the whole group agrees, or no one does it". Generally, if the leader is convinced, he goes to the group and explains what it is. If the group is not convinced, nothing is done, but if the leader has convinced the group, everyone does it. This does not imply a lack of individuality among street adolescents. On the contrary, outside of situations where their safety is threatened, requiring a united front, each adolescent is responsible for themselves and can make their own choice or decision. Our approach to this population was based on four basic principles: i) The principle of acceptance: accept the person as he or she is and do not try to make him or her fit our own criteria of normality; ii) The principle of non-judgment: do not judge, criticize or condemn; iii) The principle of universality: look at the adolescent as a whole: consider adolescents as a whole, giving them the same rights and treatment; iv) The principle of individuality: for each adolescent interviewed, take into account his or her experience and personal history and do not necessarily expect him or her to react in the same way as others.

We therefore defined the following recruitment strategy: i) Identification of sites and different groups of street adolescents; ii) Identification of group leaders; iii) Initiation of discussions with group leaders by the research team and project NGO partners; iv) Feedback from leaders to other adolescents; v) Meeting/discussion between the project team/NGO and all adolescents; vi) Verification of pre-inclusion criteria; vii) Bus transport of eligible adolescents who agreed to participate in the study from the street to the inclusion sites ("Jade pour la vie" in Lomé and "CREUSET-Togo" center in Kara).

The sample size was calculated using a single proportion population formula with a 95% confidence level. For an acceptance assumption of 80% for self-testing [22], with a precision of 5% and allowing for a 10% non-participation rate, the self-test should have been offered to at least 266 street adolescents.

In the StAdoS project, the aim went beyond the evaluation of HIV self-testing to include the provision of a sexual and reproductive health care package for street adolescents aged 13–19 years. Therefore, a primary group of street adolescents was recruited for this purpose. Given the estimated sample size for the HIV self-test evaluation and the limited availability of HIV self-tests, it was necessary to select from the primary group. Therefore, random sampling was applied to all street adolescents enrolled in the study to determine those who would be offered the HIV self-test.

### Data collection

Data collection, sampling, and HIV testing were performed at the sites of the NGOs Jade pour la vie and CREUSET-Togo. These two NGOs have health centers that provide various services, including HIV prevention, screening, and care. The administration of the questionnaire and the collection of informed consent were carried out by medical interns who were trained prior to the survey. After explaining the purpose of the study and obtaining consent or assent from the adolescents, data were collected in three stages. The first stage involved the collection of data on socio-demographic characteristics through the administration of a face-to-face questionnaire. Then, based on random sampling, selected adolescents were offered an oral HIV self-test, the OraQuick ADVANCE® Rapid HIV-1/2 Antibody Test (OraSure Technologies, Bethlehem, Pennsylvania, USA). Those who agreed to take the test were given an information session by a trained educator on how to perform a self-test. The adolescents then retired to a closed room set aside for the occasion to perform their HIV self-test without any assistance. Finally, a double reading (by street adolescent and an educator trained in HIV self-testing) of the results was carried out. A blood sample was also taken for HIV screening according to the national algorithm.

### Operational definitions

Acceptability was defined as the proportion of acceptance of oral HIV testing, where the numerator was the number of participants who tested themselves, and the denominator was number of those offered the test [23]. In this study, feasibility was defined as the participant’s ability to correctly follow the various steps to perform the test in order to obtain a valid result. The numerator was the number of tests with valid results, and the denominator was the total number of participants who performed or attempted to perform the test [23].

### Statistical analysis

Descriptive statistics were performed, and results are presented in numbers and proportions for categorical variables. HIV prevalence was estimated with its 95% confidence interval (95%CI). Quantitative variables were presented as median with interquartile range (IQR). Qualitative variables as sociodemographic characteristics and sexual behaviors were compared using the chi-squared and/or Fisher test, and the Wilcoxon test was used to compare quantitative variables as median age of adolescents and median age at first sexual intercourse. Factors associated to acceptability and feasibility of HIV self-test have been identified using two multiple weighted logistic regression models to take account of the imbalance observed in the modalities of the dependent variables. Adjusted odds ratios (aOR) were estimated with their 95% confidence intervals. A Receiver Operating Characteristic (ROC) curve was used to visualize model performance and the area under the ROC curve (AUC) provided a single scalar measure of each model’s performance. All analyses were performed using R statistical software, version 4.3.2 (R Foundation for Statistical Computing, Vienna, Austria). The statistical inference threshold was set at 5%.

### Ethical considerations

This study was approved by Togolese Bioethics Committee for Health Research (approval N°12/2020/CBRS). Participants aged 18 years and over gave informed consent. For minors (<18 years), in addition to their mandatory assent, and as recommended by the WHO in its guidelines on ethical aspects to be considered in the design and review of research on adolescent sexual and reproductive health [24], the person responsible for the adolescent (in this case the guardian within the NGO) was asked to sign the consent form. All adolescents were informed that they could withdraw from the study at any time without having to justify their decision. Adolescents who refused to participate in the study were treated in the same way as the others and were invited to attend information sessions and condom distribution at the site if they so wished.

All adolescents who tested positive for HIV were enrolled in the HIV cohort of the inclusion site, and an NGO referrer was assigned to assist them in follow-up. Adolescents with a confirmed positive HIV blood test were treated according to national recommendations.

## Results

**Fig 1:**
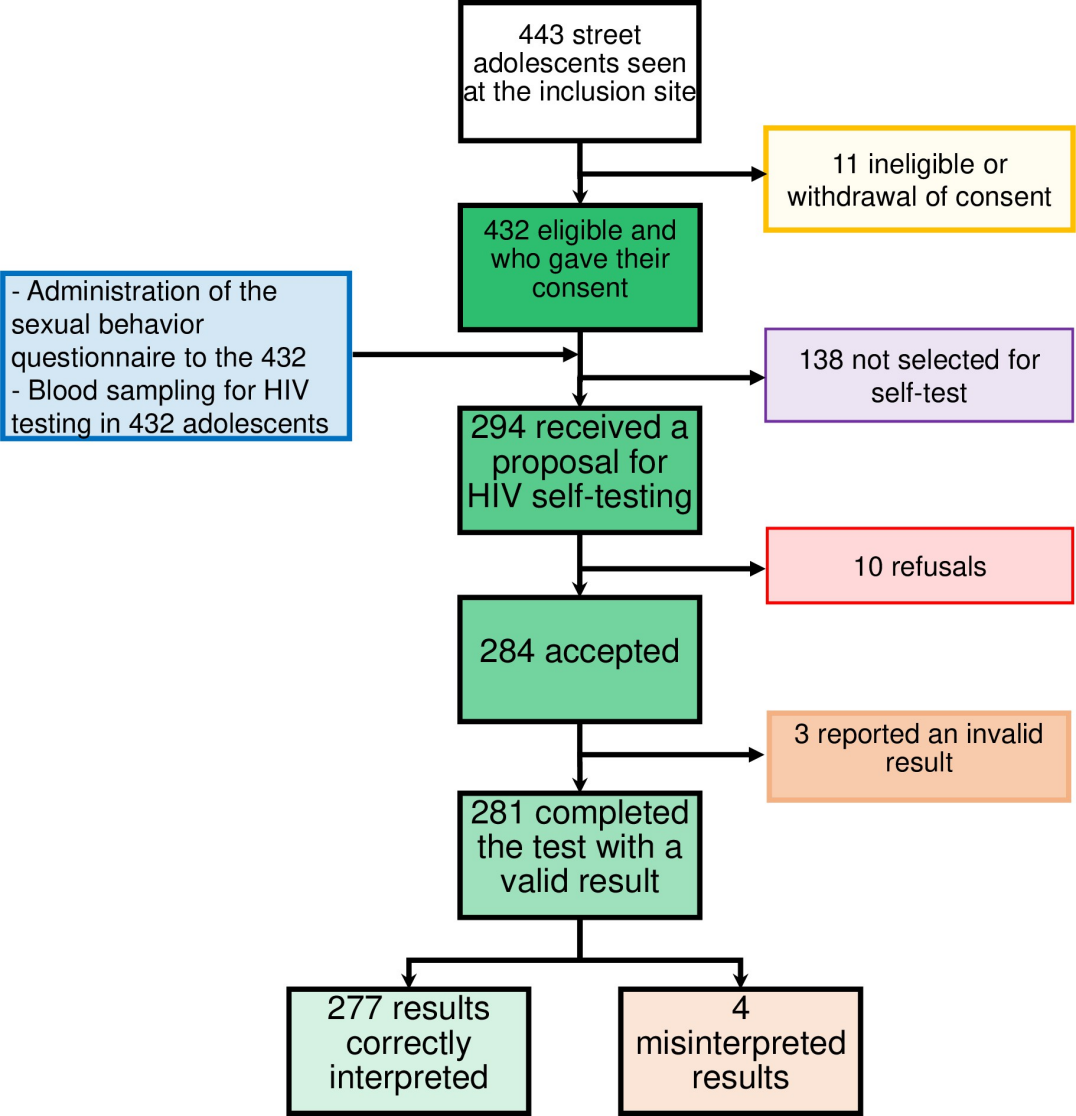
Flow chart showing the inclusion process. Fig 1 shows the flow chart and the different interventions proposed at each stage of the enrolment process.

A total of 432 street adolescents were enrolled in the study, of whom 294 (68.1%) were offered self-testing (Fig 1).

### Socio-demographic characteristics, sexual and addictive behavior of all the street adolescents

The median age of the street adolescents in Lomé and Kara was 15 years (IQR = [14–17]). About two thirds (64.4%) of the adolescents were older than 15 years. Males were the most represented, accounting for 87.7% (379/432) of the total sample (**Table 1**).

**Table 1:** Socio-demographic characteristics, sexual and addictive behavior of street adolescents according to HIV self-test selection, Togo, 2021 (N = 432).

	Selected for HIV self-test		
	No n = 138	Yes n = 294	Total N = 432
**Age (Years)**			
Median [IQR]	15 [13–17]	15 [14–17]	15 [14–17]
<15	57 (41.3)	97 (33.0)	154 (35.6)
≥15	81 (58.7)	197 (67.0)	278 (64.4)
**Gender,** n (%)			
Female	8 (5.8)	45 (15.3)	53 (12.3)
Male	130 (94.2)	249 (84.7)	379 (87.7)
**Educational level, n (%)**			
None	10 (7.2)	40 (13.6)	50 (11.6)
Primary	54 (39.1)	115 (39.1)	169 (39.1)
Secondary	57 (41.3)	114 (38.8)	171 (39.6)
University	17 (12.3)	25 (8.5)	42 (9.7)
**Nationality, n (%)**			
Togolese	120 (87.0)	257 (87.4)	377 (87.3)
Other^$^	18 (13.0)	37 (16.6)	55 (12.7)
**Sexualy active**			
No	47 (34.1)	154 (52.4)	201 (46.5)
Yes	91 (65.9)	140 (47.6)	231 (53.5)
**Age of first sexual intercourse (Years)**			
Median [IQR]	13 [10.3–15.0]	14 [12.0–16.0]	13 [12.0–16.0]
<13	38 (46.3)	42 (31.8)	80 (37.4)
13–15	28 (34.1)	48 (36.4)	76 (35.5)
≥16	16 (19.5)	42 (31.8)	58 (27.1)
Missing	9	8	17
**Number of sexual partners since the first sexual intercourse, n (%)**			
<2	32 (36.0)	47 (33.8)	79 (34.6)
2–4	32 (36.0)	51 (36.7)	83 (36.4)
≥5	25 (28.1)	41 (29.5)	66 (28.9)
Missing	2	1	3
**Use of condom during the last sexual intercourse, n (%)**			
No	65 (71.4)	96 (68.6)	161 (70.0)
Yes	26 (28.6)	44 (31.4)	70 (30.0)
**Drug abuse**			
No	80 (58.0)	220 (74.8)	300 (69.4)
Yes	58 (42.0)	74 (25.2)	132 (30.6)
**Regular alcohol consumption**			
No	39 (28.3)	149 (50.7)	188 (43.5)
Yes	99 (71.7)	145 (49.3)	244 (56.5)

IQR = Interquartile range; $Beninese, Ghanaian, Nigerian, Guinean.

Of the 432 street adolescents included in this study, 53.5% (n = 231) were sexually active. Since their first sexual experience, 28.9% (66/231) of street adolescents had had more than 5 sexual partners. Of the 231 sexually active adolescents, only 30.3% (n = 70) reported using a condom during their last sexual intercourse. In addition, 30.6% (132/432) of adolescents reported drug abuse. **Table 1** shows the socio-demographic characteristics and behaviors of street adolescents according to self-test selection.

### Socio-demographic characteristics, sexual and addictive behavior of street adolescents selected for HIV self-testing

Among adolescents selected for HIV self-testing, the proportion of girls was significantly higher in Kara than in Lomé, with 29.6% (37/125) and 4.7% (8/169) respectively. In the same group, the proportion of sexually active adolescents was significantly higher in Lomé than in Kara, with 71.6% (121/169) versus 15.2% (19/125) respectively. **Table 2** shows the socio-demographic characteristics and sexual behaviors of street adolescents selected for HIV self-testing by study site.

**Table 2:** Socio-demographic characteristics, sexual and addictive behavior of street adolescents selected for HIV self-test by study site.

	Kara, n = 125	Lomé, n = 169	Total, N = 294	p*
**Age**				
Median [IQR]	15.0 (14.0, 17.0)	16.0 (14.0, 17.0)	15.0 (14.0, 17.0)	0.200
				0.350
<15	45 (36.0)	52 (30.8)	97 (33.0)	
≥15	80 (64.0)	117 (69.2)	197 (67.0)	
** Gender, n (%)**				<0.001
Female	37 (29.6)	8 (4.7)	45 (15.3)	
Male	88 (70.4)	161 (95.3)	249 (84.7)	
**Educational level, n (%)**				0.007
None	26 (20.8)	14 (8.3)	40 (13.6)	
Primary	47 (37.6)	68 (40.2)	115 (39.1)	
Secondary	46 (36.8)	68 (40.2)	114 (38.8)	
University	6 (4.8)	19 (11.2)	25 (8.5)	
**Nationality, n (%)**				<0.001
Togolese	123 (98.4)	134 (79.3)	257 (87.4)	
Other$	2 (1.6)	35 (20.7)	37 (12.6)	
**Sexualy active**				<0.001
No	106 (84.8)	48 (28.4)	154 (52.4)	
Yes	19 (15.2)	121 (71.6)	140 (47.6)	
**Age of first sexual intercourse (Years), (n = 140)**				
Median [IQR]	17.0 [16.0–17.0]	13.0 [11.0–15.0]	13.0 [12.0–16.0]	<0.001
				<0.001
<13	0 (0.0)	42 (37.2)	42 (31.8)	
13–15	3 (15.8)	45 (39.8)	48 (36.4)	
≥16	16 (84.2)	26 (23.0)	42 (31.8)	
Missing	0	8	8	
**Number of sexual partners since the first sexual intercourse, n (%)**				0.490
<2	7 (36.8)	40 (35.1)	47 (35.3)	
2–4	9 (47.4)	42 (36.8)	51 (38.3)	
≥5	3 (15.8)	32 (28.1)	35 (26.3)	
Missing	0	7	7	
**Use of condom during the last sexual intercourse, n (%)**				0.100
No	10 (52.6)	86 (71.1)	96 (68.6)	
Yes	9 (47.4)	35 (28.9)	44 (31.4)	
**Drug abuse**				<0.001
No	120 (96.0)	100 (59.2)	220 (74.8)	
Yes	5 (4.0)	69 (40.8)	74 (25.2)	
**Regular alcohol consumption**				<0.001
No	104 (83.2)	45 (26.6)	149 (50.7)	
Yes	21 (16.8)	124 (73.4)	145 (49.3)	

*Wilcoxon rank sum test; Pearson’s Chi-squared test; Fisher’s exact test.

### Prevalence of HIV

According to the national HIV screening algorithm, four out of the 432 adolescents sampled were HIV positive, all male, giving a prevalence of 0.9% (95%CI [0.4–2.4]). Of these adolescents, three were in the city of Lomé and one in Kara. In terms of sexual activity, two of the four adolescents, aged 18 and 14, were sexually active (all in Lomé). Of the two sexually active adolescents, only one had used a condom during the last sexual encounter. Among these HIV-positive adolescents, three had benefited from HIV self-testing and all these three HIV self-tests were also reactive.

### Acceptability of HIV self-testing

Of the 294 adolescents offered the HIV self-test, 284 finally performed it, with an overall acceptability of 96.6% (95% CI = [93.8–98.3]). Acceptability was 94.1% (95% CI = [89.4–97.1]) in Lomé and 100.0% (95% CI = [97.1–100.0]) in Kara, with a significant statistical difference between the two cities (p = 0.006). Among those who declined the test, the primary reasons for refusal were: i) concern about the potential outcome (lack of reassurance) (3/10), ii) skepticism about the accuracy of the test (4/10), iii) perceived complexity of the test method (1/10), and iv) unspecified reasons (2/10).

### Feasibility of HIV self-testing

Of the 284 adolescents who performed the self-test, 281 obtained a valid result. This corresponds to an overall feasibility of 98.9% (95%CI = [97.0–100.0]). The feasibility was 98.1% (95%CI = [94.6–99.6]) in Lomé and 100% (95%CI = [97.1–100]) in Kara. The difference between the two cities was not statistically significant (p = 0.258). In addition, of the 281 tests performed, the reported result was concordant between the adolescent and the educator in 277 tests (98.6%).

### Factors associated to acceptability and feasibility

**Fig 2:**
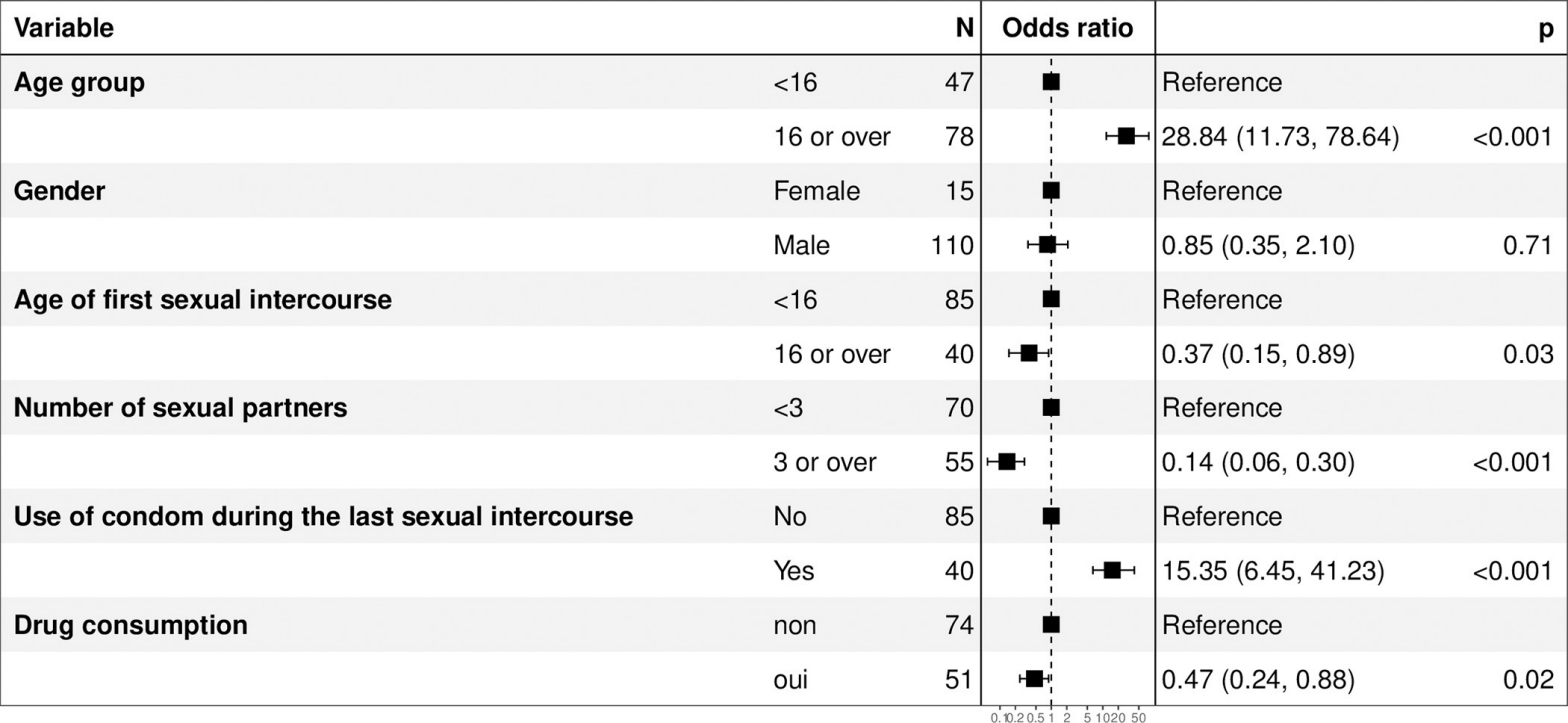
Factor associated to acceptability of HIV self-testing among street adolescents, Togo, 2021. Fig 2 presents the results of a multivariable regression model as a forest plot showing the factors associated or not with acceptability, with their adjusted odds ratios.

According to the multiple weighted logistic regression, factors associated with HIV self-test acceptability included being 16 years of age or older (aOR = 28.84; p<0.001) and having used a condom during the last sexual intercourse (aOR = 15.35; p<0.001). On the other hand, having 3 or more sexual partners (aOR = 0.14; p<0.001) and reporting drug abuse (aOR = 0.47; p = 0.020) were negatively associated to HIV self-test acceptability (Fig 2). The regression model had a good concordance index estimated at 77%.

**Fig 3:**
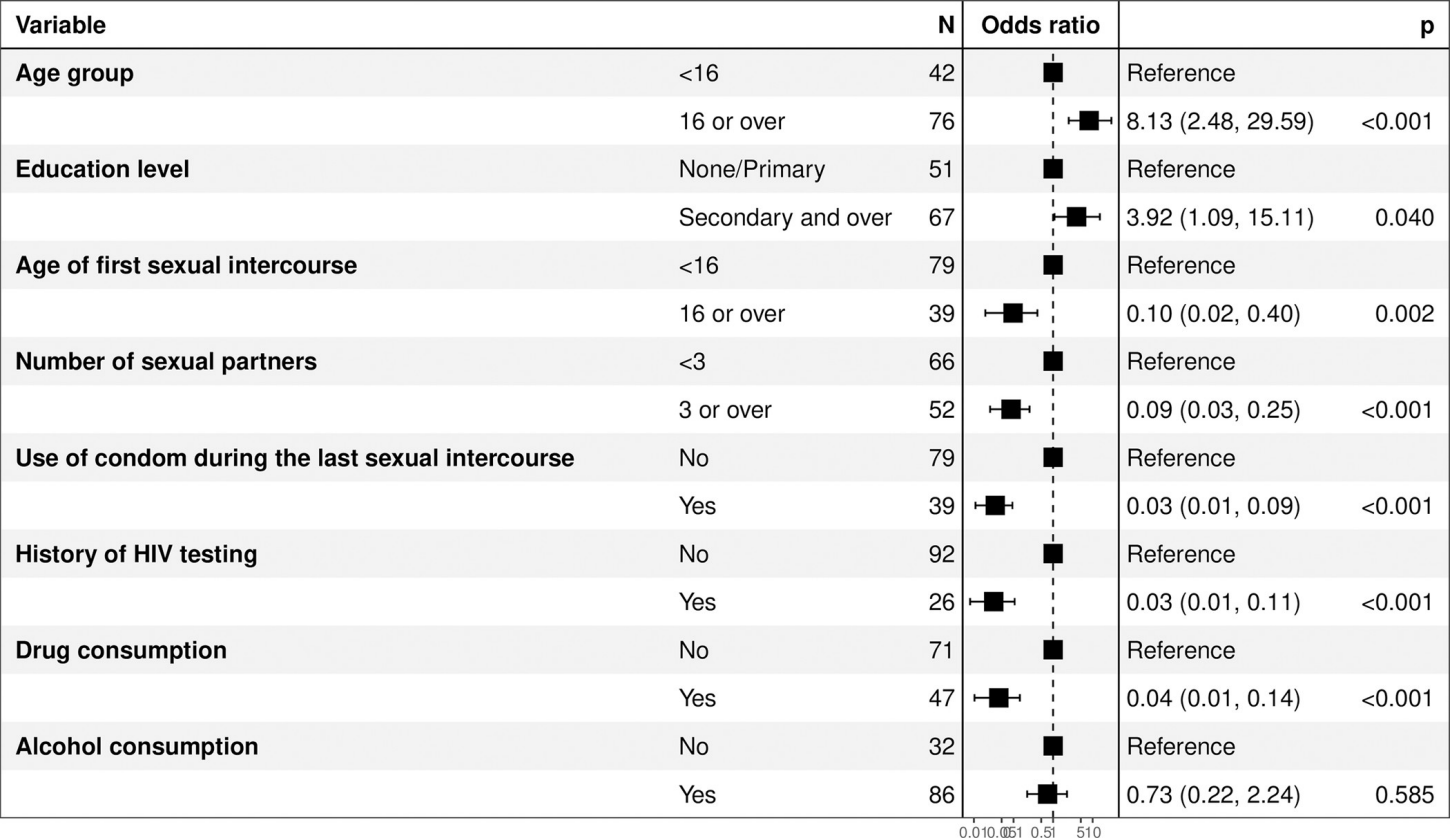
Factor associated to feasibility of HIV self-testing among street adolescents, Togo, 2021. Fig 3 presents the results of a multivariable regression model as a forest plot showing the factors associated or not with feasibility, with their adjusted odds ratios.

Factors associated to feasibility of HIV self-testing included being 16 years of age or older (aOR = 8.13; p<0.001) and education level at least equivalent to secondary school (aOR = 3.92; p = 0.040). On the other hand, condom use during the last sexual intercourse (aOR = 0.03; p<0.001), having been tested for HIV (aOR = 0.03; p<0.001), and drug abuse (aOR = 0.04; p<0.001) were negatively associated to HIV self-test feasibility (Fig 3). The regression model had a good concordance index estimated at 82%.

## Discussion

The aim of this study was to assess the acceptability and feasibility of HIV self-testing, to estimate HIV prevalence among street adolescents in Togo, and to describe the sexual behavior of this population.

More than half of the adolescents surveyed (53.5%) were sexually active at the time of the survey. The median age at first sexual intercourse in our study was 13 years (interquartile range (IQR) [12–16]). This age is relatively low compared to the median age of first sexual intercourse reported in the general population in Togo. In fact, in the last Togolese Demographic and Health Survey, the median age at first sexual intercourse was 18 years [25]. In addition, other studies report similar findings within the street adolescent population to those we found. In Ethiopia in 2018, Fikre et *al*. reported that the proportion of sexually active street adolescents was 56.1%, with a mean age of first sexual intercourse of 15.7 years [26]. In a study by Kakchapati et *al*. in Nepal, 51.5% of street adolescents surveyed were sexually active and 55% had had their first sexual intercourse before the age of 15 [27]. The reported results among the surveyed adolescents could be explained by the fact that street life exposes them to early sexual activity, which is associated with precariousness. In fact, apart from a lack of knowledge about sexual health, street adolescents may be engaged in sex in exchange for money or food [28].

In the subgroup of sexually active street adolescents, there was a large difference between Kara and Lomé (19 vs 212). One of the main reasons for this disparity could be that Lomé, as the capital city, has a larger street adolescent population [21]. Furthermore, a prevarication bias cannot be excluded for adolescents in Kara. The question on sexual activity may have been perceived as a potential source of judgment. As a reminder, the socio-cultural context of Lomé (the country’s capital) is such that people are much freer to talk about sexual matters. It should also be noted that many more girls were included in Kara than in Lomé (29.6% vs. 5.2%); in fact, boys are more likely to talk about their sexuality than girls [29].

This study also reported inconsistent condom use among street adolescents. Hartmann et *al*. in Brazil reported 61.9% of unprotected sex among street adolescents [30]; Kakchapati et *al*. in Nepal [27] and Mthembu et *al*. in Rwanda [8] reported proportions of 60.6% and 53.7% respectively for inconsistent condom use during the last sexual intercourse. The main reason for inconsistent condom use could be related to a lack of information on sexual and reproductive health or difficulty in accessing condoms in this population. This situation exposes street adolescents to unwanted pregnancies, a higher risk of HIV infection and STIs. Sexual and reproductive health programs should be specifically designed for these adolescents by leveraging the initiatives of local NGOs or by enhancing their capacity to effectively communicate for behavior change and distribute condoms.

Few studies in the literature have reported HIV prevalence among street children, particularly in Africa. In a study conducted in India in 2010, HIV prevalence among street children was estimated at 1% [31]. According to another study conducted in Nepal among 251 street children and young people, HIV prevalence was estimated at 7.6% [32]. In Iran, the prevalence of HIV in this population has been estimated at 4.5% [33]. One of the few African studies to report HIV prevalence in this population is that of Nyumayo et al. in Tanzania in 2022, which reported an HIV prevalence of 12.2% [34]. Compared with previous results, the prevalence of HIV in our study may seem low. Indeed, we reported an HIV prevalence of 0.9% in this population. However, contextual factors need to be considered, particularly the burden of HIV in the countries where the studies were conducted. In Togo, for example, the prevalence of HIV in the general population was 1.7% in 2022 [35], while in Tanzania it was 4.3% [36], context in which the Nyumayo et *al*. study was conducted. However, although a prevalence of 0.9% may seem low in comparison with other studies, it remains higher than the HIV prevalence among young men in Togo, which was 0.4% [0.4–0.6] in 2022 [35]. This result emphasizes the urgent need to ensure better access to HIV prevention and screening services for this population through innovative approaches such as HIV self-testing.

Acceptability of HIV self-testing was 96.6% among street adolescents in Togo. Although these are different contexts and populations, it should be noted that other studies have reported very high levels of acceptability. Tonen-Wolyec et *al*. in the Democratic Republic of Congo reported 95.1% acceptability among adolescents aged 15 to 19 in the city of Kisangani in 2018 [18]. In Thailand, Phongphiew et *al*. reported 87.8% acceptability among young boys who have sex with men and transgender adolescents [17].

The feasibility of HIV self-testing was 98.9%. Our results are similar to those of Tonen-Wolyec et al. in the Democratic Republic of Congo [18] and Hector et *al*. in Mozambique [16] which reported feasibility rates of 96.1% and 98.3% respectively.

Taken together, these results demonstrate that street adolescents are a target population for HIV self-testing with high levels of acceptability and feasibility. This is attributed to the non-invasive nature of the test, its practicality and the ease of sample collection, which does not involve a prick [23]. Effective and sustainable implementation of this strategy could increase access to HIV testing among street adolescents and keep them on the sexual and reproductive health care pathway.

Togo’s adoption of the HIV self-testing approach at the end of 2020, and the planned scaling-up of this strategy among key and vulnerable populations, represent real opportunities to include and offer this strategy to street adolescents [19]. To facilitate the integration of street adolescents into this strategy, the present study analyzed the factors associated with the acceptability and feasibility of HIV self-testing among this population in Togo.

Factors associated with HIV self-test acceptability included being 16 years of age or older and having used a condom during the last sexual intercourse. On the other hand, having 3 or more sexual partners and reporting drug abuse were negatively associated to HIV self-testing acceptability. In many countries, the age of 16 is the age of sexual majority, when people can make decisions about their sexual health. In our study, it appears that adolescents aged 16 and over have a greater tendency to accept HIV self-testing than those under 16. Other studies have reported the same trend, marked by an increase in testing with adolescent age. Wang et al. reported in 2017 in a survey of young people in Tanzania that younger age group was significantly associated with never testing for HIV [37]. Similar results have been reported in 2016, in other study using the Demographic and Health Survey (DHS) data from Congo (Brazzaville), Mozambique, Nigeria, and Uganda [38]. Authors reported that, older youth had significantly higher odds of ever being tested for HIV than younger respondents [38]. Substance abuse was also reported as negatively associated to HIV self-test acceptability in the present study. This finding is in line with the literature, which reports poorer adherence and limited access to HIV testing among substance abusers [39,40].

Factors associated with feasibility of HIV self-testing included being 16 years of age or older and education level at least equivalent to secondary school. This result could be explained by a better ability to observe and follow test instructions in older and more educated adolescents. Furthermore, a high level of education is known to be associated with high levels of HIV testing. In a 2014 study of women aged 15–49 in Zambia, educational level was associated with higher HIV testing in 15,388 women of childbearing age [aOR 3.8, 95% CI 1.7–8.2; p = 0.001] [41]. In another study published in 2022 and based on data from the DHS survey done among 11 east African countries, age (highest) and educational level, were significantly associated with HIV testing [42]. On the other hand, condom use during the last sexual intercourse, having been tested for HIV, and drug abuse were negatively associated with HIV self-test feasibility. The negative association between condom use at last sexual intercourse and previous HIV screening with feasibility could be linked to a low perceived risk or a false sense of security, which may have led to less attention being paid to the instructions for the HIV self-test. In fact, perceived risk is recognized in the literature as a factor associated with better adherence to and use of screening services [43].

To implement this strategy among street adolescents effectively and achieve high levels of acceptability and feasibility, it is important to consider several key elements. When introducing HIV self-testing among street adolescents, it is essential to integrate a program to prevent drug abuse and develop a communication strategy about HIV risk and self-testing. The messages should be tailored to reach the youngest and least educated adolescents. Ensuring a good understanding of the HIV self-testing strategy and implementation procedures will lead to better adherence to the test, accurate results, and correct interpretation of these results by adolescents.

A misinterpreted HIV test can lead to a false sense of security or unwarranted anxiety. Therefore, regulatory validation studies of screening tests also focus on the user’s ability to correctly interpret the result. For the OraQuick HIV self-test used in this study, the proportion of correctly interpreted results in the FDA evaluations was 93.9%, 92.6% and 90.8% for positive, negative and invalid results, respectively [44]. In the present study, HIV self-test results were correctly interpreted by 98.6%, reflecting a low risk of false sense of security or unjustified anxiety among street adolescents who perform the test.

This study is the first to provide data on the acceptability and feasibility of HIV self-testing among street adolescents in Togo. However, it has certain limitations. The first limitation is that 138 street adolescents were not selected for self-testing because of sample size and HIV self-test availability constraints. A selection bias cannot be excluded; in fact, the non-inclusion of these adolescents may have overestimated or underestimated acceptability and feasibility of HIV self-test. Secondly, a prevarication or social desirability bias cannot be excluded due to the sensitivity of some of the questions raised in the survey, in particular drug abuse, which was purely declarative and for which no pharmacological tests were performed.

## Conclusion

Street adolescents in Togo are a vulnerable population for HIV and STIs, with early initiation of sexual activity and inconsistent condom use. Access to conventional screening remains limited for this population. The present study reports that HIV self-testing is acceptable and feasible among street adolescents and could be a game changer for this population, particularly in a context such as Togo, where self-testing is already implemented for key populations. The provision of self-testing kits, coupled with condom distribution through outreach centers, could help to improve access to HIV care services. For this approach to gain wide acceptance among street adolescents, it is essential to integrate a drug abuse prevention program. Additionally, continuous communication is necessary, with a particular focus on those under 16 and those with lower educational levels. A tailored communication strategy should be developed specifically for these groups.
